# Blockade of programmed death-1/programmed death ligand pathway enhances the antitumor immunity of human invariant natural killer T cells

**DOI:** 10.1007/s00262-016-1901-y

**Published:** 2016-09-15

**Authors:** Toshiko Kamata, Akane Suzuki, Naoko Mise, Fumie Ihara, Mariko Takami, Yuji Makita, Atsushi Horinaka, Kazuaki Harada, Naoki Kunii, Shigetoshi Yoshida, Ichiro Yoshino, Toshinori Nakayama, Shinichiro Motohashi

**Affiliations:** 1 Department of Medical Immunology, Graduate School of Medicine, Chiba University, 1-8-1 Inohana, Chuo-ku, Chiba, 260-8670 Japan; 2 Department of General Thoracic Surgery, Graduate School of Medicine, Chiba University, 1-8-1 Inohana, Chuo-ku, Chiba, 260-8670 Japan; 3 Department of Otorhinolaryngology, Head and Neck Surgery, Graduate School of Medicine, Chiba University, 1-8-1 Inohana, Chuo-ku, Chiba, 260-8670 Japan; 4 Department of Immunology, Graduate School of Medicine, Chiba University, 1-8-1 Inohana, Chuo-ku, Chiba, 260-8670 Japan

**Keywords:** Invariant NKT cells, Anti-PDL1 antibody, PD-1, Antitumor immunity

## Abstract

The role of invariant natural killer T (iNKT) cells in antitumor immunity has been studied extensively, and clinical trials in patients with advanced cancer have revealed a prolonged survival in some cases. In recent years, humanized blocking antibodies against co-stimulatory molecules such as PD-1 have been developed. The enhancement of T cell function is reported to improve antitumor immunity, leading to positive clinical effects. However, there are limited data on the role of PD-1/programmed death ligand (PDL) molecules in human iNKT cells. In this study, we investigated the interaction between PD-1 on iNKT cells and PDL on antigen-presenting cells (APCs) in the context of iNKT cell stimulation. The blockade of PDL1 at the time of stimulation resulted in increased release of helper T cell (Th) 1 cytokines from iNKT cells, leading to the activation of NK cells. The direct antitumor function of iNKT cells was also enhanced after stimulation with anti-PDL1 antibody-treated APCs. According to these results, we conclude that the co-administration of anti-PDL1 antibody and alpha-galactosylceramide (αGalCer)-pulsed APCs enhances iNKT cell-mediated antitumor immunity.

**Electronic supplementary material:**

The online version of this article (doi:10.1007/s00262-016-1901-y) contains supplementary material, which is available to authorized users.

## Introduction

Invariant natural killer T (iNKT) cells are innate-like immune cells, characterized by their invariant T cell receptor. They respond to glycolipid antigens, such as α-galactosylceramide (αGalCer), presented by the HLA class I-like molecule CD1d, and rapidly produce substantial amounts of cytokines upon stimulation [1]. iNKT cells play an important role in antitumor immunity via the activation of antigen-presenting cells (APCs), T cells, and natural killer (NK) cells as well as displaying direct cytotoxic functions toward cancer cells [2–4]. It is reported that the number of iNKT cells in the peripheral blood decreases and that proliferation in response to its ligand is impaired in cancer patients [5, 6]. Translational research aimed at manipulating the antitumor functions of iNKT cells is ongoing, and clinical benefits have been reported in some patients with non-small cell lung cancer (NSCLC) or head and neck cancer. The clinical benefits of the administration of αGalCer-pulsed APCs are especially seen in patients with improved cytokine production in response to its ligand following therapy. However, many patients with advanced cancer respond poorly to this therapy and the survival outcomes of these patients remain poor [2, 7, 8]. This is, in part, attributed to the immunosuppressive tumor microenvironment that abrogates effective antitumor immunity, and strategies to overcome the immune suppression are needed to eradicate the tumor and improve the prognosis [9–12].

Programmed death-1 (PD-1) is a co-receptor that is expressed on activated lymphocytes. It is reported to regulate the function of activated T and B cells to induce peripheral tolerance, induce immunological memory, and prevent excess tissue damage in cases of infection [13]. Its ligands, PDL1 (B7-H1) and programmed death ligand 2 (PDL2, B7-DC), are expressed on professional APCs and many tumor cells. PD-1/PDL interaction in the induction phase and effector phase of antitumor immunity is one of the mechanisms utilized by tumors to avoid T cell-mediated rejection [14, 15]. Recent clinical trials of the systemic administration of anti-PD-1 or anti-PDL1 antibody have shown objective and durable responses in patients with NSCLC, melanoma, and renal cell cancer [16, 17]. At the same time, severe adverse events have been reported, including adrenal insufficiency, myocarditis [16], pneumonitis, and hepatitis [17].

There have been a few reports on the role of PD-1/PDL1 in iNKT cells. In murine tumor models, the blockade of PD-1/PDL1 interaction led to enhanced interferon gamma (IFNγ) secretion and anti-metastatic activity of iNKT cells [18–20]. The function of PD-1/PDL on human iNKT cells has been poorly studied. The expression of PD-1 is reported to be elevated in the iNKT cells of tuberculosis and HIV patients. However, the reported impact of PD-1/PDL blockade on iNKT cell function differed among the studies [21–23]. We therefore focused on the inhibitory effects of PD-1/PDL interaction and assessed whether the blockade of this pathway could improve the antitumor responses of ligand-activated iNKT cells. Results from our study showed that the surface expression of PD-1 was upregulated on activated human iNKT cells and blockade of PDL1 on αGalCer-pulsed APCs augmented Th1 cytokine production and direct cytotoxic function of iNKT cells. Furthermore, the upregulation of Th1 cytokines resulted in increased NK cell-mediated cytotoxicity. From these findings, we concluded that the administration of APCs that are preincubated with anti-PDL1 antibodies would enhance iNKT cell-mediated antitumor immunity and provide the basis for new clinical studies on the administration of iNKT cell-mediated immunotherapy with immune checkpoint inhibitors.

## Materials and methods

### Cell lines

The human lung cancer cell line NCI-H460 [H460] (ATCC^®^ HTB-177™) was purchased from American Type Culture Collection (ATCC). K562 cells were kindly provided by Dr. C. H. June [24]. Jurkat and A549 cells were kindly provided by Dr. K. Suzuki [25]. All cell lines were cultured in RPMI complete medium supplemented with 10 % FBS and antibiotics.

### Antibodies and reagents

Recombinant human IL-2 (Imunace; Shionogi, Osaka, Japan), recombinant human IFNγ (R&D), human GM-CSF (GeneTech Co. Ltd, China) and α-galactosylceramide (αGalCer, KRN7000; REGiMMUNE, Tokyo, Japan) were used for cell culture and stimulation. For plate-bound assays, 96-well flat bottom culture plates were purchased from Greiner Bio-One. Recombinant soluble dimeric human CD1d:Ig fusion protein was purchased from BD Biosciences. Recombinant human PDL1 Fc chimera fusion protein and IgG_1_ Fc, and human anti-CD80 antibody (clone #37711) were purchased from R&D. Anti-human CD28 antibody (clone 28.2) was purchased from BioLegend. For anti-PDL1 antibody removal, Dynabeads^®^ M-280 Sheep anti-Mouse IgG was used together with DynaMag™-2 Magnet (both were purchased from Novex and Life Technologies). Twelve-well plates with 0.4 μm polycarbonate membrane inserts (Corning) were used for the transwell assays.

FITC-conjugated anti-TCR Vα24 antibody (clone C15) and PE Vβ11 (clone C21) was purchased from Beckman Coulter. Functional grade and allophycocyanin (APC)-conjugated anti-human PDL1 antibodies (clone 29E.2A3), anti-PDL2 (clone MIH18), APC anti-human PD-1 (clone EH12.EH7), PE anti-human PDL2 (clone 24F.10C12), anti-human CD80, APC/cyanin 7 (Cy7) anti-human CD3 (clone HIT3a), and matching isotype controls were each purchased from BioLegend. PE anti-human CD3 (clone HIT3a), FITC anti-human CD56 (clone NCAM16.2) and APC anti-human CD56 were purchased from BD Pharmingen. Biotin-SP-conjugated AffiniPure goat anti-mouse IgG,F(ab’)_2_ fragment-specific antibody was purchased from Jackson ImmunoResearch Laboratories and used together with APC streptavidin (BD Pharmingen) to assess blocking antibody binding in cultured cells. Peroxidase streptavidin (Jackson ImmunoResearch) was used in an ELISA to determine the concentration of anti-PDL1 antibodies. For cell purification, anti-FITC MicroBeads and the NK Cell isolation kit, CD56 microbeads, and CD3 microbeads were purchased from Miltenyi Biotec. Human IFNγ, IL-4, and TNFα ELISA sets (BD), the BD Cytometric Beads Array (CBA) System (BD), Bio-Plex Pro Human Cytokine 27-Plex Panel (Bio-Rad), and the Milliplex MAP Human CD8^+^ T Cell Magnetic Bead Panel kit (Merck Millipore) were used for cytokine detection. Cytotoxicity assays were performed with the Cytotoxicity Detection Kit^PLUS^ (LDH) from Roche.

### Collection of peripheral blood samples

Peripheral blood was obtained from healthy donors and NSCLC patients after obtaining their informed consent. All experiments were performed in accordance with the Declaration of Helsinki and approved by our institutional review board (#1016).

### Preparation of antigen-presenting cells (APCs)

Peripheral blood mononuclear cells (PBMCs) were isolated via density gradient separation using Ficoll-Paque™ PLUS and cultured in RPMI 1640 complete medium with 100 JRU/ml of IL-2 and 800 U/ml of GM-CSF, as previously described [26]. For the cytotoxicity assays and cytokine secretion assays, CD56-positive cells were depleted using the autoMACS^®^ Pro Separator on day 0. A total of 200 ng/ml of αGalCer was added on day 6, and all cultured cells were collected on day 7. The cultured cells underwent 50 Gy of radiation before being added to the iNKT cell culture.

### Induction of in vitro activated iNKT cells

PBMCs were cultured with antibody-treated αGalCer-pulsed APCs and IL-2 for 7 days. On days 0 and 7, cells were counted and the percentage of Vα24^+^Vβ11^+^ cells and the rates of PD-1 and PDL1 positivity were assessed by flow cytometry using the FACSCanto™ II analyzer and FlowJo software program. For proliferation assays, the cells were restimulated on day 7 with antibody-treated αGalCer-pulsed APCs. On days 7 and 14, the number of iNKT cells was determined by multiplying the total cell count by the percentage of Vα24^+^Vβ11^+^ cells based on the flow cytometric data.

### Preparation of αGalCer-pulsed plate-bound CD1d

Flat bottom 96-well plates were coated with 0.5 μg of CD1d dimeric protein with 2 μg of recombinant human PDL1 Fc chimera fusion protein or IgG_1_ Fc and 2 μg of anti-CD28 agonistic antibody in 30 μl PBS. After incubation for 6 h at 4 °C, 50 ng of αGalCer was added and the plate was incubated overnight at 37 °C. The next day, the wells were washed with PBS and incubated with cell culture medium before the addition of antibodies and NKT cells.

### Cytokine secretion assay

Cultured iNKT cells were purified on day 7 via positive selection of Vα24-FITC-stained cells and cultured overnight in medium supplemented with 100 JRU/ml of IL-2. On day 8, the cells were collected and cultured in 96-well plates. A total of 2 × 10^5^ iNKT cells (1 × 10^5^ in patients) were stimulated with plate-bound CD1d pulsed with αGalCer or 1 × 10^6^ antibody-treated αGalCer-pulsed APCs. In the plate-bound assays, iNKT cells were treated with anti-PD-1 antibody, anti-CD80 antibody, or isotype control at 20 μg/ml prior to stimulation. The supernatants were collected after 24 h, and cytokine concentrations were assessed by ELISA. Multiplex cytokine assays were performed with the Bio-Plex Pro Human Cytokine Assay kit. The Bio-Plex 3D system was used for the analysis.

### Cytotoxicity assay

For the iNKT cell-mediated cytotoxicity assays, Vα24^+^ cells were positively selected on day 7, as described above. On day 8, the cells were counted and incubated with cancer cells at an E/T ratio of 5:1–15:1 in 96-well plates. Prior to co-culture, the cancer cells were suspended in medium and incubated for 30 min with 10 μg/ml of anti-PDL1 antibody or isotype-matched control. After 4 h of co-culture, cytotoxicity was assessed by measuring LDH release. The supernatants were collected, and the release of cytotoxic molecules was analyzed using the Milliplex MAP Human CD8^+^ T Cell Magnetic Bead Panel kit.

For NK-mediated cytotoxicity assays, the cells were purified using an NK Cell Isolation Kit, and NK cells were cultured for 24 h in supernatant obtained from αGalCer-pulsed APC-restimulated iNKT cells. Prior to NK cell stimulation, Sheep anti-Mouse IgG beads were applied to the supernatant, incubated for 30 min, and then magnetically removed together with the captured anti-PDL1 antibody or isotype control proteins. The cytokine concentrations in the supernatants were measured with the BD™ Cytometric Bead Array kit and FACS Verse analyzer. After 24 h, the NK cells were collected, counted, and co-cultured with cancer cells in 96-well plates at an E/T ratio of 2:1. The degree of cytotoxicity and levels of cytotoxic molecules were assessed as described above. In the assays with direct cell contact, NK cells were cultured for 24 h with NKT cells and αGalCer-pulsed APCs, after which they were purified by the depletion of CD3 positive cells and positive selection with CD56 microbeads. The outcomes of stimulation with soluble factors were compared to those of direct cell–cell contact using 0.4-μm-pore transwells with NK cells in the lower chamber and NKT cells plus αGalCer-pulsed APCs in the upper chamber.

### Statistical analysis

The statistical analysis was performed using the GraphPad PRISM^®^ software program version 6.0b. Error bars represent the standard deviation in all graphs. *p* values of <0.05 were considered to be statistically significant.

## Results

### PD-1 expression on human iNKT cells

**Fig. 1:**
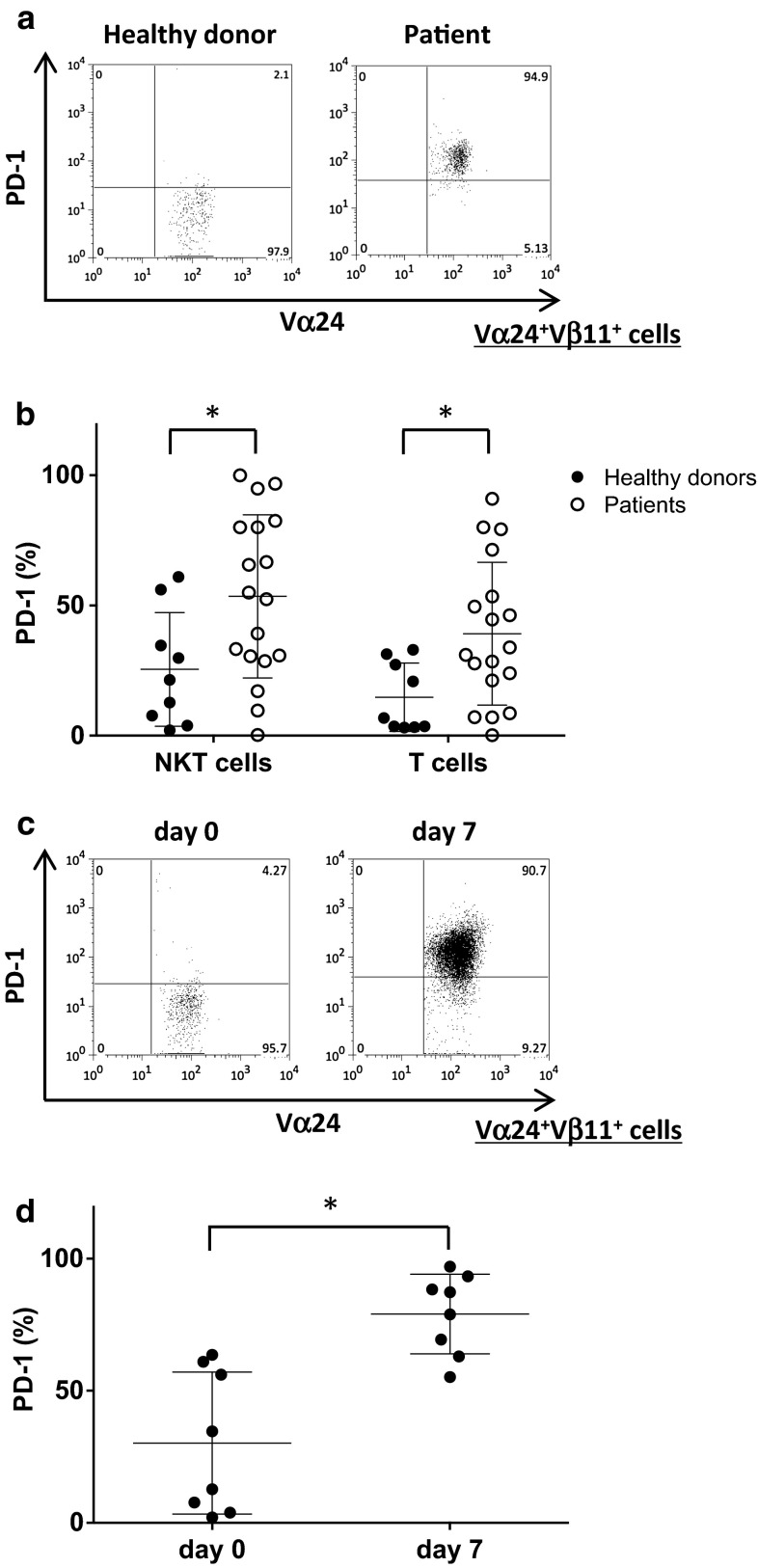
PD-1 expression on human iNKT cells. **a** Representative FACS profiles of the PD-1 expression on Vα24^+^Vβ11^+^ iNKT cells obtained from healthy donors and patients. **b** The proportions of PD-1^+^ cells among Vα24^+^Vβ11^+^ iNKT cells and CD3^+^ T cells obtained from healthy donors (*black circle*) and patients (*open circles*) were determined by flow cytometry. The results are presented as the mean values with the distribution. *p < 0.05 (healthy controls vs. patients, unpaired *t* test). **c**, **d** PBMCs were obtained from eight healthy donors. Fresh PBMCs were stimulated with αGalCer-pulsed APCs with anti-PDL1 blocking antibody or isotype control antibody on day 0. **c** Representative profile of the PD-1 expression in Vα24^+^Vβ11^+^ iNKT cells before culture and 7 days after stimulation. **d** The proportions of PD-1^+^ cells among Vα24^+^Vβ11^+^ iNKT cells obtained from healthy donors before and 7 days after stimulation are depicted. *p < 0.05 (unpaired *t* test)

PBMCs were obtained from nine healthy donors and 18 NSCLC patients. All patients were diagnosed with unresectable advanced or recurrent NSCLC. Freshly isolated healthy donor-derived peripheral blood iNKT cells expressed low levels of PD-1. In contrast, PD-1 expression on iNKT cells and T cells obtained from NSCLC patients was significantly higher than that observed in healthy volunteers (Fig. 1a, b). Next, we evaluated the changes in PD-1 expression on in vitro activated iNKT cells derived from healthy donors. The percentage of PD-1 positive iNKT cells increased following stimulation with αGalCer (Fig. 1c, d). According to these results, we hypothesized that PD-1/PDL1 blockade on αGalCer-pulsed APCs at the time of iNKT cell stimulation could improve iNKT cell function.

### Proliferative response of iNKT cells stimulated with PDL1 blocked APCs

**Fig. 2:**
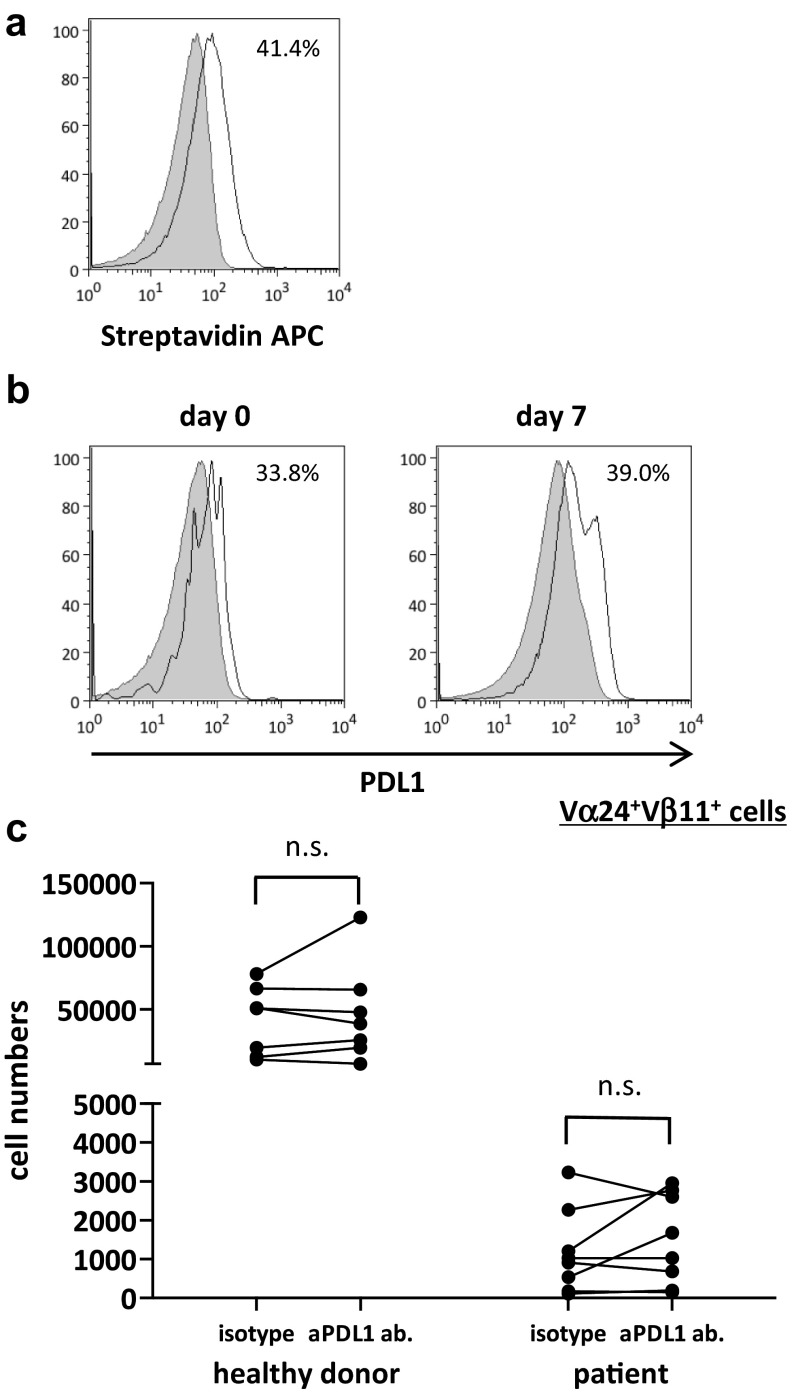
Proliferation of human iNKT cells with PDL1 blockade. PBMCs were obtained from six healthy donors and eight non-small cell lung cancer patients. On day 0, PBMCs were stimulated with αGalCer-pulsed IL-2/GM-CSF cultured APCs with anti-PDL1 antibody or isotype control. On day 7, cells were collected and restimulated with PDL1-blocked or isotype control-treated APCs at a ratio of 1:2.5. The cells were collected and counted on day 14, and the proportion of Vα24^+^Vβ11^+^ iNKT cells was analyzed using flow cytometry. **a** Anti-PDL1 antibody binding and PDL1 positivity on APCs were assessed using anti-mouse biotin plus streptavidin staining. **b** The percentage of PDL1-positive iNKT cells on days 0 and 7 were analyzed with APC-conjugated anti-human PDL1. The *gray-shaded* histogram represents the isotype control; the *unshaded* histogram represents PDL1. **c** The number of Vα24^+^Vβ11^+^ iNKT cells on day 7 is shown. PDL1 positivity on APCs was analyzed according to the population comparison method using the FlowJo software program. P values were calculated using the unpaired *t* test. isotype, isotype control; aPDL1 ab, anti-PDL1 antibody

To investigate the role of anti-PDL1 antibodies in the proliferative responses of αGalCer-pulsed APC-stimulated iNKT cells, αGalCer-pulsed APCs were preincubated with anti-PDL1 or control antibody before addition to iNKT cell culture on days 0 and 7 (Fig. 2a). PDL1 was expressed on iNKT cells as well as on the APCs (Fig. 2b). Although the number of iNKT cells stimulated with anti-PDL1 antibody-treated APCs tended to increase in both healthy donors and patients, the results differed widely among the donors with no significant differences between the two groups (Fig. 2c). The application of anti-PDL1 antibodies could not reverse the impaired proliferative function found in the cancer patients to the level of healthy subjects.

### Cytokine production of iNKT cells stimulated in the presence of PDL1

**Fig. 3:**
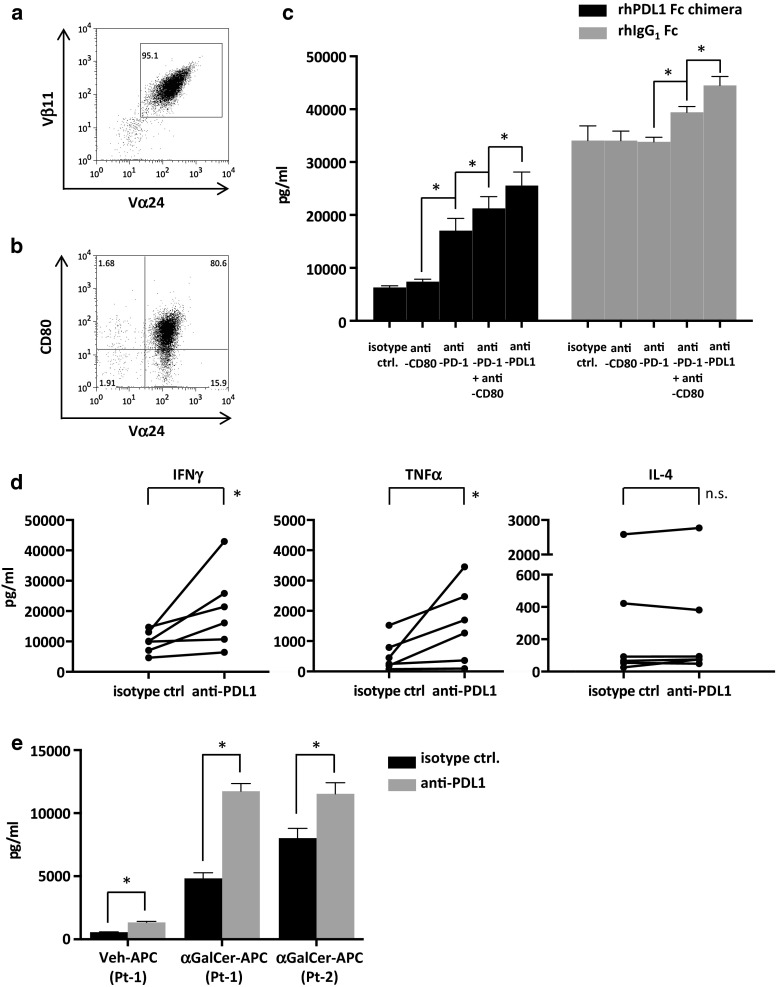
Cytokine secretion of iNKT cells following restimulation. PBMCs obtained from healthy donors were stimulated on day 0 with αGalCer and IL-2. On day 7, Vα24-FITC-stained cells were purified by autoMACS Pro Separator positive selection. **a** The expanded cells were analyzed via FACS to determine the proportions of iNKT cells using anti-Vα24 and anti-Vβ11 antibodies. A representative result of assays performed with six donors (day 7 after MACS separation) is shown. **b** CD80 and Vα24 positivity in the purified iNKT cells used in the assay. **c** αGalCer-pulsed CD1d and recombinant human PDL1 fusion protein or control protein-coated 96-well flat bottom plates with anti-CD28 agonistic antibody were used to stimulate the purified iNKT cells on day 8. Anti-PDL1 antibody, anti-PD-1 antibody, anti-CD80 antibody, or matched isotype control were added before stimulation. Culture supernatants were collected at 24 h, and cytokine concentration assays were performed by ELISA. Stimulations were performed in quadruplicates. Statistical analysis was performed using the unpaired *t* test. *p < 0.05. rhPDL1 Fc chimera, recombinant human PDL1 Fc chimera fusion protein; rhIgG_1_, recombinant human IgG_1_. **d** Purified healthy donor-derived iNKT cells at 2 × 10^5^ cells were restimulated with 1 × 10^6^ anti-PDL1 antibody or isotype control-treated APCs in 96-well plates. The culture supernatants were collected at 24 h, and ELISA of the cytokine concentrations were performed. Statistical analysis was performed using the paired *t* test. *p < 0.05. **e** A total of 1 × 10^5^ purified iNKT cells obtained from advanced lung cancer patients were restimulated as described above with αGalCer or vehicle-pulsed APCs, preincubated with either anti-PDL1 antibody or isotype control. The IFNγ concentration in the supernatants was analyzed as described above. A statistical analysis was performed using the unpaired t-test. *p < 0.05. isotype ctrl., isotype control; anti-PDL1, anti PDL1 antibody; anti-PD-1, anti-PD-1 antibody; anti-CD80, anti-CD80 antibody; Veh-APC, vehicle-pulsed APC; αGalCer- APC, αGalCer-pulsed APC; Pt, patient

iNKT cells obtained from healthy donors were cultured with αGalCer and IL-2. On day 7, the cells were stained with anti-Vα24 FITC and positively selected via the autoMACS Pro Separator. The expression levels of CD80 as well as PD-1 were upregulated in the cultured iNKT cells that were used in the assay (Fig. 3b).

Because PDL1 is reported to interact with both PD-1 and CD80 [27], the isolated cells were collected on day 8 and incubated with anti-PD-1 antibody, anti-CD80 antibody, both antibodies, or isotype control. αGalCer-pulsed CD1d and PDL1 fusion protein or IgG_1_ control protein pre-coated 96-well plates were incubated with anti-PDL1 antibody or matching isotype control, and iNKT cells at 2 × 10^5^ cells per well were seeded. The culture supernatants were collected at 24 h, and the IFNγ concentration was measured with ELISA.

The stimulation of iNKT cells by recombinant human PDL1 fusion protein resulted in a decrease in the production of IFNγ in comparison with the control protein. The blockade of PD-1 on iNKT cells resulted in a significant improvement in IFNγ release. The blockade of both PD-1 and CD80 resulted in a threefold increase, while the blockade of PDL1 led to further enhancement (Fig. 3c). From these results, we concluded that the presence of PDL1 fusion protein had a regulatory role on iNKT cell cytokine production through interactions with both PD-1 and CD80. There was also a minor but significant increase in the production of cytokines in the control protein plus anti-PDL1 antibody or anti-PD-1 plus CD80 groups, which may result from the interaction with the PDL1 expressed on the activated iNKT cells. Since the anti-PDL1 antibody used in the assay appeared to block interaction with both PD-1 and CD80, we mainly focused on the role of anti-PDL1 antibody in iNKT cell activation.

### Cytokine production of iNKT cells with PDL1-blocked APCs

We further evaluated the cytokine production of iNKT cells when stimulated with APCs as performed in clinical settings. iNKT cells obtained from healthy donors were cultured with antibody-treated APCs. On day 7, Vα24^+^ cells were purified as described above and restimulated on day 8 with matched APCs. The culture supernatant was collected after 24 h, and the cytokine concentrations were measured with ELISA. Results showed increased IFNγ and TNFα levels in the PDL1-blocked APC group, whereas there were no significant changes in the levels of IL-4 (Fig. 3d). Similar trends were observed with multiplex assays. IFNγ, TNFα, GM-CSF, and IL-2 levels tended to be increased in the PDL1-blocked APC group, whereas there were no significant changes in the levels of IL-4, IL-13, IL-5, or IL-9 (Supplemental Fig. S1). These results indicate that blocking the PD-1/PDL1 interaction promotes Th1 cytokine secretion in activated iNKT cells. Since the lymphocytes from advanced cancer patients are reported to express multiple inhibitory factors [28], similar cytokine secretion assays were performed with iNKT cells derived from advanced lung cancer patients. Although the percentage and proliferation were extremely low in comparison with healthy donors, the iNKT cell purity was at 50–75 % after magnetic separation on day 7. IFNγ secretion was significantly increased to a level that was comparable to that in healthy donors, when anti-PDL1 antibodies were applied (Fig. 3e).

### Changes in the cytokine profiles with the addition of anti-PDL2 antibody

**Fig. 4:**
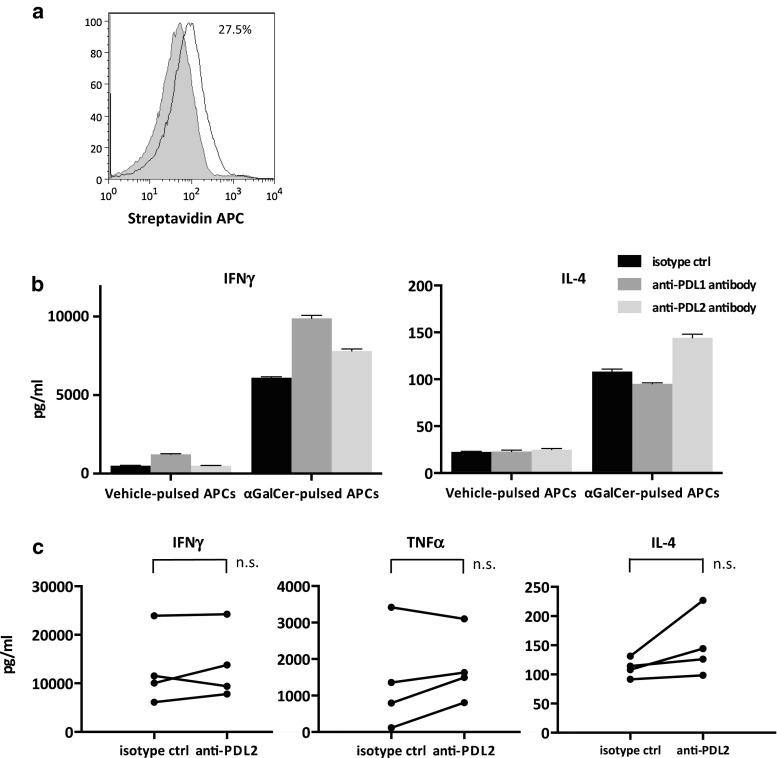
The impact of PDL2 blockade on cytokine secretion by activated iNKT cells. Fresh PBMCs derived from healthy donors were stimulated as in Fig. 3. APCs were preincubated with anti-PDL2, anti-PDL1, or isotype control antibody before addition to the culture. iNKT cells were purified and restimulated as in Fig. 3, and the supernatants were collected after 24 h. **a** PDL2 positivity and antibody binding on APCs were assessed as in Fig. 2a. The *gray-shaded* histogram represents the isotype control, and the *unshaded* histogram represents PDL2. The IFNγ, IL-4, and TNFα concentrations were analyzed using ELISA. **b** Representative data for one healthy donor are presented. **c** The results of the comparison between PDL2-blocked and isotype control systems in four donors are shown. Statistical analyses were performed using the paired *t* test. isotype ctrl, isotype control; anti-PDL2, anti-PDL2 antibody

Akbari et al. [29] previously reported that PDL2 regulates Th2 cytokine production in mouse iNKT cells, which subsequently contributes to airway hypersensitivity. Because IL-2/GM-CSF-cultured APCs also expressed low levels of PDL2 in this study (Fig. 4a), we evaluated the potential of iNKT cells to produce various cytokines after stimulation with anti-PDL2 antibody-treated APCs. PDL2 blockade on APCs resulted in little increase in the Th1 cytokine levels compared to that observed in the PDL1-blocked APCs. On the other hand, IL-4 production tended to increase in the supernatants following anti-PDL2 antibody-treated APC stimulation; however, it did not reach statistical significance (Fig. 4b, c).

### iNKT cell-mediated cytotoxicity toward tumor cell lines

**Fig. 5:**
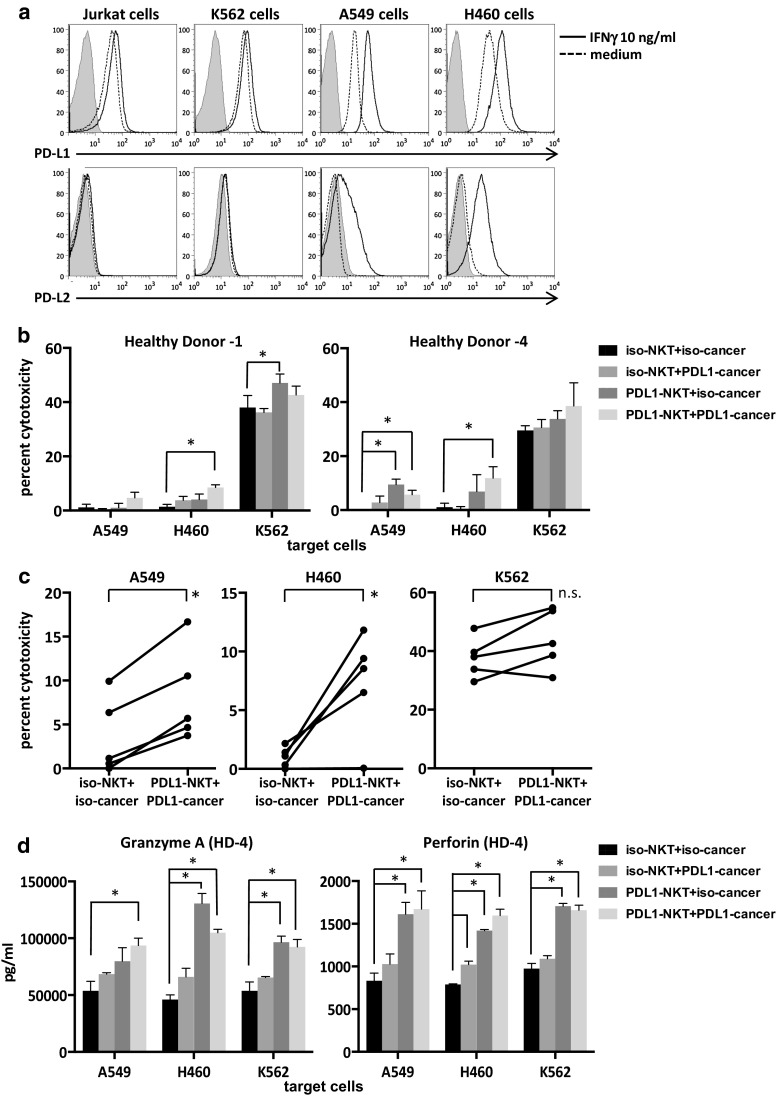
PDL1 expression in cancer cell lines and iNKT cell-mediated cytotoxicity. **a** Cancer cell lines were cultured for 24–48 h in RPMI1640 complete medium alone or with 10 ng/ml of recombinant human IFNγ. PDL1 and PDL2 positivity was determined using flow cytometry. *Shaded histograms*: isotype control, *dashed lines*: medium only, *bold lines*: cultured with IFNγ. To test for cytotoxicity, iNKT cells were cultured and purified as in Fig. 3. On day 8, iNKT cells were incubated with the tumor cell lines at an E/T ratio of 15:1 for 4 h. The percent cytotoxicity was determined based on the degree of LDH release according to the manufacturer’s protocol. The assays were performed in triplicate with cells obtained from two donors. **b** Representative values for two to four independent experiments for each donor. The *error bars* represent the standard deviation. *P* values were calculated using multiple *t* tests. **c** The results of the cytotoxicity assays performed with iNKT cells derived from five donors at an E/T ratio of 5:1–15:1. *p* values were calculated with paired *t* tests. **d** The supernatants of cells incubated as in **b** were collected, and multiplex assays were performed in duplicate to determine the concentrations of cytotoxicity molecules. Cells were obtained from the same donors as in (**b**). Statistical analysis was performed as in (**b**). The graphs with *error bars* show the standard deviation. *p < 0.05. iso-NKT, iNKT cells stimulated with isotype control plus APC on day 0; PDL1-NKT, iNKT cells stimulated with PDL1-blocked APCs on day 0; iso-cancer, cancer cells incubated with isotype control antibody before the cytotoxicity assay; PDL1-cancer, cancer cells incubated with anti-PDL1 antibody before the cytotoxicity assay; HD, healthy donor

Since direct cytotoxicity is one of the important functions of activated iNKT cells, we evaluated the role of anti-PDL1 antibodies in the direct cytotoxic activities of iNKT cells against several tumor cell lines, including lung cancer. The PDL1 and PDL2 expression levels in four tumor cell lines, including Jurkat, K562, A549 and H460, were analyzed by flow cytometry. PDL1 was expressed in all four cell lines. The expression of PDL1 was upregulated after IFNγ exposure in the cancer cell lines (Fig. 5a). PDL2 expression was very low in all four cell lines, and the expression levels increased in the two lung cancer cell lines with IFNγ. In order to assess whether PDL1 blockade would improve iNKT cell-mediated cytotoxicity, we cultured iNKT cells with anti-PDL1 antibody-treated APCs starting on day 0 and performed 4-h cytotoxicity assays with purified iNKT cells on day 8. The iNKT cells stimulated with anti-PDL1 antibody-treated APCs showed increased cytolysis of CD1d-negative lung cancer cell lines and K562 in two healthy donors (Fig. 5b). Similar experiments were performed in three additional donors at E/T ratios of 5:1–15:1. Invariant NKT cells cultured with PDL1-blocked APCs displayed significantly increased cytolysis toward PDL1-blocked lung cancer cell lines in comparison with the isotype controls (Fig. 5c). Multiplex assays of supernatants revealed increased levels of perforin and granzyme A from iNKT cells stimulated with anti-PDL1 antibody-treated APCs in healthy donor 4 (Fig. 5d). Similar trends were obtained in the samples obtained from healthy donor 1.

### Adjuvant effects on NK cell-mediated immunity via activated iNKT cells

**Fig. 6:**
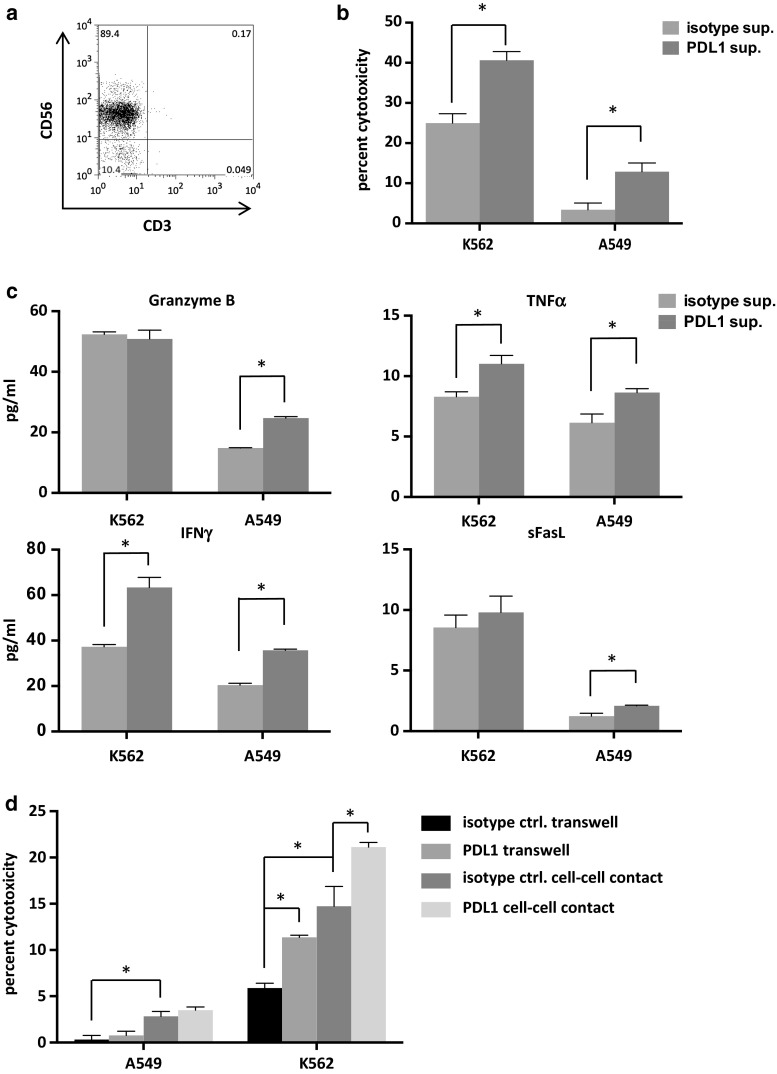
Induction of NK cell-mediated cytotoxicity via activated iNKT cells. iNKT cells were purified and restimulated as in Fig. 3d. The supernatants were collected 6 h after restimulation, and residual antibodies were magnetically removed with the addition of Dynabeads^®^ Sheep anti-Mouse IgG. **a** NK cells were obtained from freshly isolated PBMCs of a healthy donor by autoMACS Pro Separator negative selection using a NK isolation kit. NK cells were cultured in iNKT cell supernatants at a concentration of 7.5 × 10^5^ cells/ml. After 24 h, the NK cells were collected, washed, counted, and seeded into 96-well plates with the cancer cell lines. The E/T ratio was 2:1 for K562 cancer cells and A549 cancer cells. **b** The degree of cytotoxicity at 4 h in quadruplicate and **c** the levels of released cytotoxic molecules in duplicate were determined as in Fig. 5. **d** NK cells were seeded in the lower compartment of a 0.4-μm-pore transwell plate. PDL1-blocked or isotype control APCs and iNKT cells were added at a ratio of 1:1:1 in either the upper compartment or the lower compartment. Twenty-four hours later, the cells from the lower chamber were collected and anti-CD3 beads were applied. Following negative selection via MACs, the CD3-negative cells were incubated with anti-CD56 beads and NK cells were positively selected. The cytotoxicity assays were performed as described above. The error bars represent the standard deviation. Isotype sup: NK cells cultured in supernatant derived from iNKT cells stimulated with isotype control antibody plus APCs, PDL1 sup: NK cells cultured in supernatant derived from iNKT cells stimulated with anti-PDL1 antibody plus APCs. Transwell: NK cells stimulated with iNKT cells and APCs in the upper compartment of a transwell system, cell–cell contact: NK cells stimulated with iNKT cells and APCs in the same compartment, sFasL: soluble Fas ligand. *p < 0.05 (multiple *t* tests)

Stimulation of iNKT cells with PDL1-blocked APCs resulted in the increased production of IL-2 and IFNγ. In order to examine whether soluble factors derived from activated iNKT cells could affect NK cell functions, we collected iNKT cell culture supernatants and magnetically removed anti-PDL1 antibodies with Sheep anti-Mouse IgG. In the preliminary experiments, the residual antibody concentration was as low as 0.008 %. Isolated peripheral blood NK cells (Fig. 6a) were cultured in the supernatants for 24 h, after which cytotoxicity assays against K562 and A549 tumor cells were performed. The supernatant from iNKT cells stimulated with PDL1-blocked APCs had a positive impact on NK cell-mediated cytotoxicity compared to the isotype control-treated APCs (Fig. 6b). These findings may have been, in part, due to the increased degranulation of soluble molecules such as granzyme B (Fig. 6c). To further assess the effects of PDL1 blockade in a setting where activated iNKT cells had direct contact with NK cells, we cultured iNKT cells and αGalCer-pulsed APCs together with purified NK cells at a ratio of 1:1:1 for 24 h. To compare the effects of soluble factors alone in this setting, we also cultured the cells using a transwell system. The NK cells were purified from this system with the depletion of CD3-positive cells and the positive selection of CD56 positive cells via MACS. NK cells stimulated with direct cell contact displayed enhanced cytotoxicity toward A549 and K562 cells in comparison with soluble factors alone. In K562 cells, the blockade of PDL1 on APCs resulted in improved NK cell cytotoxicity when they were stimulated with either direct cell contact or soluble factors. Although similar trends were observed in the assays of the A549 cell lines, the results did not reach statistical significance (Fig. 6d).

Taken together, these results imply that blockade of PD-1/PDL1 interaction enhances the Th1 function of iNKT cells and augments both direct and indirect antitumor immunity.

## Discussion

PD-1 was originally described as a receptor leading to apoptotic cell death of activated T cells [30]. From further research, PD-1 expression was found in activated T cells, B cells, NK cells, and NKT cells. PD-1 is reported to regulate the threshold, strength, duration, and properties of antigen-specific immunological responses [13]. Tumors utilize this system to escape immunosurveillance. The expression of PDL1 on tumor cells induces T cell death, resulting in the evasion of host immunity [30]. PD-1 is now thought to regulate T cell functions through the inhibition of CD28-mediated activation of phosphatidylinositol 3-kinase (PI3 K). PI3 K activation promotes cell survival and cytokine production in T cells [31]. In NK cells, the PI3 K pathway is involved in target cell lysis and regulates perforin and granzyme B mobilization and redistribution [32]. Moreover, in murine models, the interaction between PD-1 and PDL1 has been shown to reduce the proliferation and production of cytokines such as IFNγ in iNKT cells.

In the current study, we found an elevated PD-1 expression in freshly isolated circulating iNKT cells in cancer patients, and this expression level increased after stimulation with αGalCer, since PD-1 is expressed on activated lymphocytes. The high positivity of PD-1 in iNKT cells from cancer patients is thought to be due to prolonged stimulation by tumor-derived ligands. To test the reversibility of anergy in these iNKT cells, we should perform cytotoxicity assays against autologous tumor cells. This was practically difficult due to the low frequency of iNKT cells in PBMCs and also because the amount of available tumor cells was insufficient.

The blockade of PD-1 on iNKT cells resulted in increased IFNγ secretion when stimulated in the presence of PDL1. The mechanism underlying this observation may include the blockade of PD-1-mediated inhibition of PI3 K, although the functions of CD28 on iNKT cells are considered to be slightly different from those in T cells [33]. In addition, we also found that blockade of both CD80 and PD-1 or PDL1 resulted in higher levels of cytokine release from iNKT cells compared to PD-1 blockade alone. These results suggest that not only PD-1, but also CD80 can interact with PDL1, whose interaction negatively regulates iNKT cell function.

iNKT cells produce substantial amounts of both Th1 and Th2 cytokines upon stimulation, playing an important role in allergies, autoimmunity, infection, and antitumor immunity. The exact mechanisms as to how the immune response is regulated toward the Th1 or Th2 direction are poorly understood. Different subsets of iNKT cells may produce different cytokines or different signals, and stimulants may lead to different responses after stimulation [34]. In the present study, the blockade of PDL1 upon initial stimulation appeared to augment the release of cytotoxic molecules following tumor recognition. Additional blockade of PDL1 expressed on tumor cell lines resulted in enhanced direct cytotoxicity of the stimulated iNKT cells. Since iNKT cells are reported to not directly exhibit antibody-dependent cellular cytotoxicity (ADCC) [35], we concluded that PDL1 blockade enhances cytotoxic functions of iNKT cells. In addition, we found that anti-PDL1 antibody only improved Th1 cytokine production, whereas PDL2 blockade appeared to increase IL-4 secretion. Signals transmitted via PDL1 and PDL2 may regulate human iNKT cells toward a Th1 or Th2 profile; this process may occur through the proliferation of a certain subset of iNKT cells. This finding is similar to the observations noted in a murine model of airway hypersensitivity reaction (AHR). PDL2-deficient mice produce more IL-4 from iNKT cells, resulting in severe airway hypersensitivity. On the other hand, PDL1 deficiency or blockade results in an increase in IFNγ production and lower levels of AHR. According to these results, PDL1 and PDL2 appear to regulate mouse iNKT cell-mediated AHR in opposing directions [29]. Since both PDL1 and PDL2 bind to PD-1 and suppress PI3 K activation, the mechanisms underlying this difference may be due to the effects of other receptors, such as CD80 [36].

iNKT cell-derived cytokines, such as IFNγ and IL-2, are reported to activate cytotoxic T lymphocytes (CTL) and NK cells, which effectively abrogate the actions of tumor cells. The Th2 cytokine IL-4, on the other hand, suppresses CTL function [37] and stimulates a subset of B cells, thereby causing allergies. IL-10 is thought to induce regulatory T cells [34]. In the current study, supernatants derived from iNKT cells stimulated with PDL1-blocked APCs tended to enhance the effector function of NK cells. This may be the result of the increased ability of iNKT cells to produce Th1 cytokines such as IL-2 and IFNγ. The ability to stimulate NK cells was further enhanced by direct cell–cell contact, which is probably the result of the expression of co-stimulatory molecules on iNKT cells plus the effects of PDL1 blockade.

In this study, it was possible to enhance IFNγ production from patient-derived iNKT cells as well as healthy donors. A previous study on iNKT cell cytokine secretion in HIV patients revealed that the administration of anti-PDL1 antibody could not improve the iNKT cell functions in HIV patients [23]. In the study, the authors stimulated whole PBMCs with αGalCer instead of purifying iNKT cells, which may have led to the difference in the results. Although our patients had stage IV lung cancer, there is also the possibility that there are differences in the levels of exhaustion and functional impairment between cancer and HIV patients. Further cytokine assays with an increased number of patient donors and the analysis of other inhibitory molecules should be performed to clarify this point. It was not possible to perform cytotoxicity assays with patient-derived iNKT cells due to their low positivity and proliferation capacity. In spite of this limitation, our results show that PDL1 blockade can reverse the impaired cytokine secretion of iNKT cells in cancer patients, which may lead to the enhanced activation of NK cells and T cells and the improved cytotoxicity of these cells.

Taken together, the addition of anti-PDL1 antibodies to αGalCer-pulsed APCs enhances the effector function of iNKT cells and also recruits other effector cells toward antitumor immunity. The finding that iNKT cells may be regulated to Th1 or Th2 phenotypes may also lead to the development of new strategies for controlling autoimmunity, allergies, and transplant rejection. Further studies in vitro and in vivo are warranted to clarify the underlying mechanisms and test the feasibility of this new therapy.

## Conclusion

Blockade of the PD-1/PDL1 axis in iNKT cells enhanced both direct and indirect antitumor immunity.

## Electronic supplementary material

Below is the link to the electronic supplementary material.

Supplementary material 1 (PDF 109 kb)

## Abbreviations

AHR: Airway hypersensitivity reaction
APC: Allophycocyanin
APC/Cy7: Allophycocyanin/cyanin 7
αGalCer: Alpha-galactosylceramide
iNKT cells: Invariant natural killer T cells
NSCLC: Non-small cell lung cancer
PDL: Programmed death ligand
Th: Helper T cell
