# Supplementary material for: Blockade of programmed death-1/programmed death ligand pathway enhances the antitumor immunity of human invariant natural killer T cells
Source: Cancer Immunol Immunother. 2016 Sep 15;65(12):1477–89. doi: 10.1007/s00262-016-1901-y (PMC5099366; doi:10.1007/s00262-016-1901-y)
Supplement: Supplementary file 1 — Supplementary material 1 (PDF 109 kb) [file 262_2016_1901_MOESM1_ESM.pdf]

Supplemental Figure S1

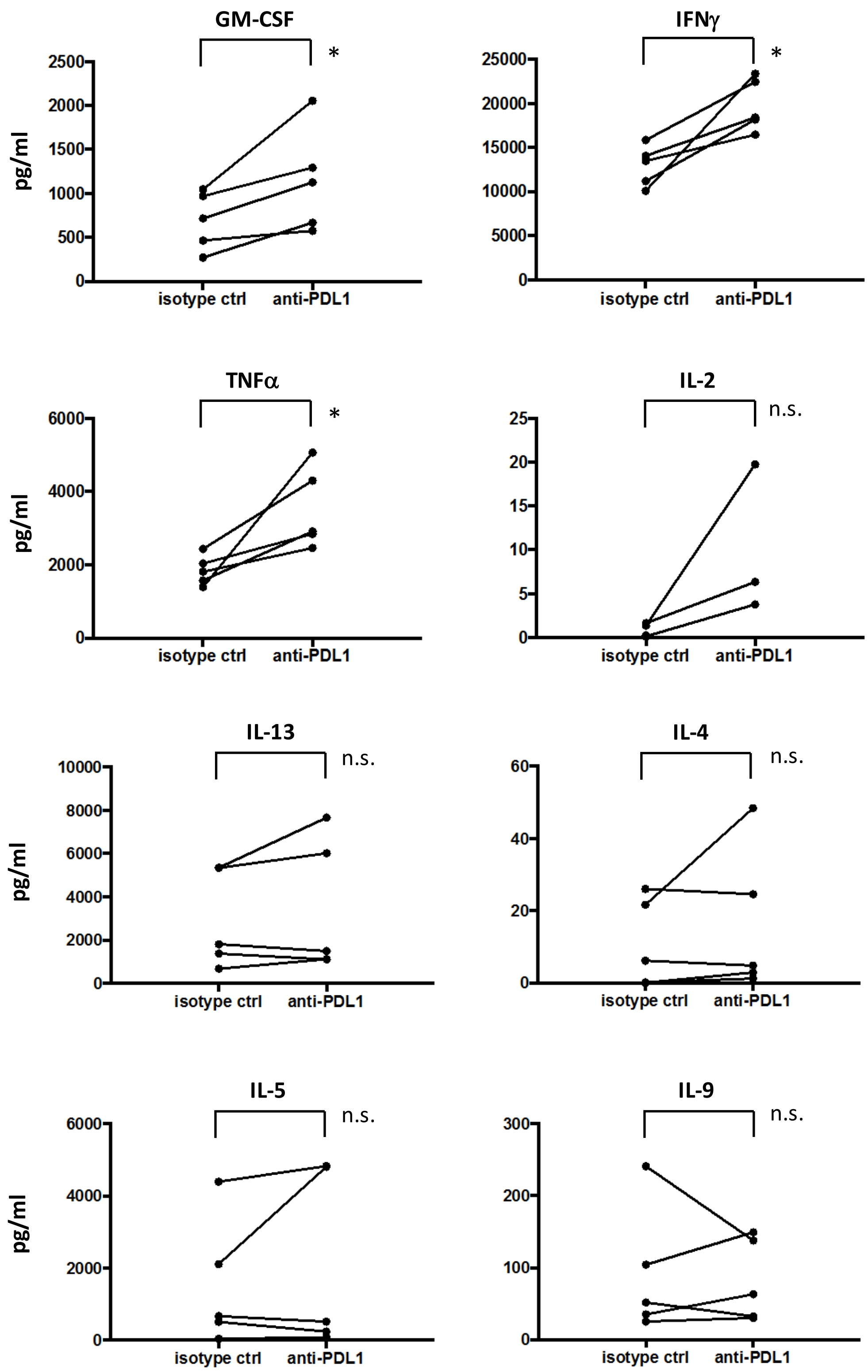

Supplemental Figure S1. Multiplex assay of cytokine secretion following iNKT cell restimulation with PDL1-blocked APCs.

PBMCs from healthy donors were stimulated with αGalCer-pulsed APCs with anti-PDL1 antibody or isotype control. On day 7, iNKT cells were purified and $2x10^5$ cells were stimulated with $1x10^6$ APCs the following day. Supernatants were collected after 24 hours and Multiplex assays were performed. P values were calculated by paired t tests. *: $p<0.05$. isotype ctrl: isotype control, anti-PDL1: anti-PDL1 antibody.
